# A Peroxymonosulfate-Based CMP Slurry for Efficient and Stable Polishing of Single-Crystal Diamond over a Wide pH Range

**DOI:** 10.3390/mi17060643

**Published:** 2026-05-23

**Authors:** Jia Chen, Tao Wu, Ping Zhou

**Affiliations:** Key Laboratory for Precision and Non-Traditional Machining Technology of Ministry of Education, School of Mechanical Engineering, Dalian University of Technology, Dalian 116024, China; 20201044012@mail.dlut.edu.cn (J.C.); 15308266852@163.com (T.W.)

**Keywords:** single-crystal diamond, chemical mechanical polishing, material removal rate, peroxymonosulfate, pH, slurry

## Abstract

Achieving efficient and high-quality surface processing of single-crystal diamond (SCD) remains challenging due to its extreme hardness and chemical inertness. Traditional Fenton-based slurries using H_2_O_2_ suffer from poor stability, safety risks, and strict acidic pH requirements. In this study, peroxymonosulfate (PMS) is introduced as an alternative oxidant to develop a novel chemical mechanical polishing (CMP) slurry for SCD. Compared with H_2_O_2_, PMS exhibits higher stability and generates sulfate radicals (SO_4_^−^·) with stronger oxidation capability when activated by Fe^2+^. The proposed slurry achieves efficient material removal over a wider pH range (2–6). Under optimal conditions (pH = 3), a maximum material removal rate (MRR) of 676 nm/h is obtained, along with an ultra-smooth surface (Sa = 0.177 nm in the measuring area of 868 × 868 μm^2^). Notably, the slurry maintains high MRR (>400 nm/h) even under weakly acidic conditions (pH 5–6). XPS and radical quenching experiments confirm that continuous generation of reactive radicals promotes surface oxidation and stable material removal. This work provides a stable and efficient CMP slurry for SCD with enhanced pH adaptability.

## 1. Introduction

Single-crystal diamond (SCD) possesses exceptional physicochemical properties, including ultra-high thermal conductivity, wide bandgap, high carrier mobility, and outstanding chemical stability [1,2,3,4]. Owing to these advantages, SCD has been widely regarded as an ideal material for next-generation high-power electronics, optical devices, and quantum technologies. However, the extremely high hardness and chemical inertness of diamond also make it one of the most difficult materials to process with high efficiency and low damage. For diamond-based electronic and optical devices, the surface quality of the substrate has a decisive influence on the surface performance and device reliability. Surface defects, subsurface damage and nanoscale roughness can significantly deteriorate the carrier transport characteristics and optical properties. Therefore, the realization of an ultra-smooth and low-damage surface is very important for the practical application of SCD [5,6,7].

At present, several processing techniques are used for diamond and related hard, brittle material surface finishing, including mechanical polishing [8], thermochemical polishing [9], plasma etching [10], laser-assisted grinding [11] and chemical mechanical polishing (CMP) [12]. Among them, CMP is considered to be the most effective method to prepare ultra-smooth surfaces because of its combination of chemical oxidation and mechanical removal [13,14,15]. In CMP, the oxidant induces surface oxidation or hydroxylation, after which abrasive particles interact with the modified surface and remove it mechanically [16]. However, the strong chemical inertness of diamond severely limits the chemical reaction rate in the CMP process, resulting in a relatively low material removal rate (MRR). Previous studies have shown that the MRR of traditional diamond CMP systems is typically below 300 nm/h, which cannot meet the growing demand for high-efficiency and high-quality diamond processing [17,18,19,20,21]. To enhance the oxidation ability of CMP slurries, various oxidants have been investigated, including KMnO_4_, K_2_Cr_2_O_7_, Na_2_S_2_O_3_, and H_2_O_2_ [21,22,23,24]. Among these oxidants, the Fenton reaction system based on H_2_O_2_ and ferrous ions (Fe^2+^) has attracted extensive attention [25,26,27] because it can generate highly reactive hydroxyl radicals (·OH) [28,29]. These radicals exhibit strong oxidation capability, which can oxidize the carbon atoms on the surface of diamond to form oxygen-containing functional groups such as C–O and C=O, thereby weakening the C–C bond and facilitating material removal during CMP [25,26].

Despite its advantages, the Fenton slurry has several limitations. First, hydrogen peroxide is thermodynamically unstable and is prone to decomposition during storage and transportation, which causes safety problems in industrial applications [30]. Second, the classical Fenton reaction is highly dependent on strongly acidic conditions (typically pH = 3), and its efficiency decreases rapidly as the pH increases due to catalyst hydrolysis and ineffective decomposition of H_2_O_2_ [31,32]. Furthermore, such a strongly acidic medium causes severe corrosion damage to polishing equipment, pipes, and mechanical components, shortening service life and raising maintenance costs. These problems seriously restrict stable polishing performance over a wide pH range and limit the practical application of the traditional Fenton polishing slurry.

In recent years, advanced oxidation technology based on sulfate radical (SO_4_^−^·) has attracted wide attention due to its strong oxidation ability and high stability [33,34]. In particular, peroxymonosulfate (PMS) is a stable oxidant that often exists in the form of solid powder (Oxone). When its aqueous solution is activated by transition metal ions such as Fe^2+^, in addition to producing a small amount of ·OH, it can also produce a large amount of SO_4_^−^· [35,36]. Compared with the hydroxyl radical, the sulfate radical has higher redox potential (2.5–3.1 V) and longer lifetime (30–40 μs), which enhances its effective oxidation ability in water systems [37]. Moreover, PMS exhibits higher chemical stability and broader pH adaptability compared with H_2_O_2_, making it a promising oxidant for developing more stable oxidation systems [34,38].

Inspired by these advantages, SO_4_^−^· is introduced into the CMP process of SCD in this study. By employing PMS activated by Fe^2+^, a CMP slurry with a broader pH processing window is developed. In this slurry, PMS is activated by Fe^2+^ to generate SO_4_^−^· and ·OH, thereby enhancing the oxidation of the diamond surface and promoting the subsequent mechanical removal process of abrasive particles. X-ray photoelectron spectroscopy (XPS) was used to clarify the oxidation mechanism of PMS + FeSO_4_ slurry on single-crystal diamond. MRRs and roughness obtained via SCD polishing using slurry based on PMS and H_2_O_2_ at different pH values are compared to evaluate the polishing stability of the proposed CMP slurry over a wide pH range. Free radical quenching experiments confirm that there are still a large number of active free radicals in the PMS + FeSO_4_ slurry in the weakly acidic environment and they play an important role in the CMP of SCD.

## 2. Experimental Details

### 2.1. Materials

**Figure 1:**
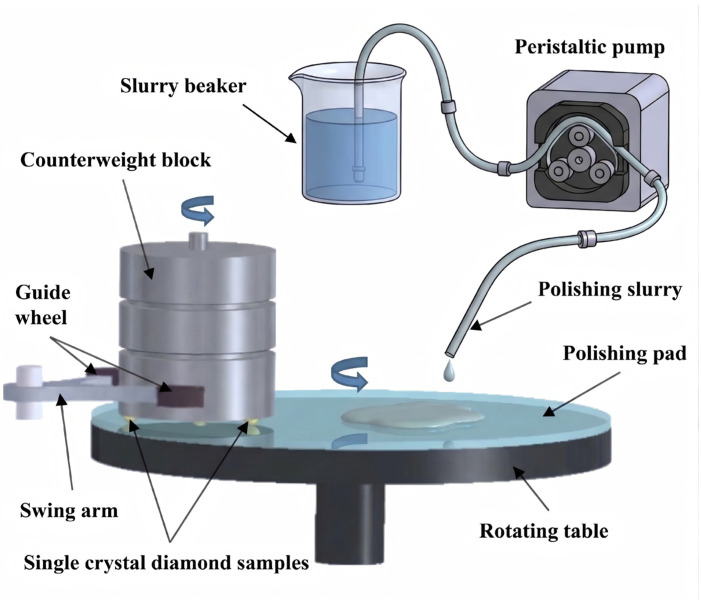
Schematic diagram of the CMP experimental setup for SCD.

The SCD (100) samples used in this study, with dimensions of 3 mm × 3 mm × 1 mm, were provided by Dongguan Jinfeng Diamond Co., Ltd. (Dongguan, China) and synthesized via the CVD method. The diagram of the experimental setup is shown in Figure 1. During the CMP process, three SCD samples were bonded along the edge of a stainless steel carrier with a diameter of 105 mm using an acid–base-resistant epoxy adhesive. The CMP experiments were conducted on an automatic precision lapping and polishing machine UNIPOL-1202 via Shenyang Kejing Auto-instrument Co., Ltd. (Shenyang, China), utilizing a smooth JGS2 quartz glass as the polishing pad. The polishing pad has a diameter of Φ300 mm and a thickness of 5 mm. Additionally, the stainless steel carrier was positioned by a bracket with guide wheels, and the polishing load was adjusted by changing the mass blocks placed on the carrier.

### 2.2. CMP Slurry Preparation

Two types of CMP slurries were prepared using diamond powder as the abrasive, PMS (42%~46% KHSO5 basis) and H_2_O_2_ as oxidants, and deionized water. Because the slurries only contain H, O, K, S, and Fe elements, H_2_SO_4_ and KOH were used as pH adjusters to formulate the CMP slurries at different pH values to avoid introducing other elements. The diamond powder used is w0.25 (particle size 0–0.25 μm), and the mass fraction is 5 wt%. The mass fractions of the oxidants in the two slurries were 5 wt% PMS and 30 wt% H_2_O_2_, respectively. The specific compositions of each CMP slurry are listed in Table 1. To ensure uniform distribution of the abrasive particles, a magnetic stirrer was continuously used to agitate the CMP slurry, and the prepared CMP slurries were subjected to 10 min of ultrasonic shock treatment before polishing to improve the dispersibility of the abrasive particles.

### 2.3. Oxidation, CMP and Radical Quenching Experiments

First, to elucidate the oxidation behavior of the PMS + FeSO_4_ system on SCD, static oxidation experiments were conducted. The SCD samples were immersed in the PMS + FeSO_4_ slurry for 12 h, followed by XPS analysis to characterize the surface chemical states after oxidation.

Then, the CMP experiments were carried out using a UNIPOL-1202 precision automatic grinding and polishing machine, with a smooth JGS2 quartz glass pad selected as the polishing pad. The experimental parameters were set as follows: polishing pressure was 0.8 MPa, polishing plate rotation speed was 80 rpm and the duration was set to 2 h. The pH values of the two groups of polishing slurry as shown in Table 1 were adjusted to 2, 3, 4, 5, and 6 to investigate the effect of pH on polishing performance. During polishing, magnetic stirring ensured uniform dispersion of abrasives, and the polishing slurry was ultrasonically treated for 10 min in advance to optimize particle dispersion. After polishing, the diamond samples sequentially underwent the following cleaning steps: first, ultrasonic cleaning in absolute ethanol for 10 min, followed by ultrasonic cleaning in deionized water for 10 min, and finally, drying with a nitrogen gun.

Finally, to verify that the PMS + FeSO_4_ CMP slurry retains its high material removal capability for SCD at pH 6 due to the presence of abundant active radicals, a radical quenching experiment was carried out by adding ethanol (EtOH) into the slurry. EtOH is widely recognized as an efficient scavenger for both ·OH and SO_4_^−^·. When EtOH is present in the reaction system, these active radicals are rapidly consumed, thereby suppressing radical-driven oxidation reactions [39]. Since the experiments were carried out at room temperature, EtOH cannot react directly with SCD. This eliminates the influence of the reaction between the scavenger itself and SCD on the MRR. By adding a certain amount of radical scavenger into the slurry, the scavenger can react directly with the active radicals generated in the slurry, thereby largely avoiding the reaction between radicals and the workpiece surface. The equations for the reactions between EtOH and different free radicals are shown in Equations (1) and (2) [39].

SO_4_^−^· + CH_3_CH_2_OH → ·CH(CH_3_)OH + SO_4_^2−^ + H^+^ (1)

·OH + CH_3_CH_2_OH → ·CH(CH_3_)OH + H_2_O (2)

The pH of the PMS + FeSO_4_ slurry was adjusted to 6. Then, 0.2 mol/L EtOH was added to the slurry as a radical scavenger. For comparison, a control experiment without EtOH was also performed under identical polishing conditions. The CMP process parameters, including polishing pressure (0.8 MPa), polishing pad speed (80 rpm), and abrasive concentration, were kept the same as those described above. Then, we measured the MRR and Sa after polishing. Sa represents the arithmetical mean height of the surface and is defined as the arithmetic average of the absolute height deviations from the mean plane within the evaluation area [40].

### 2.4. Characterization

**Figure 2:**
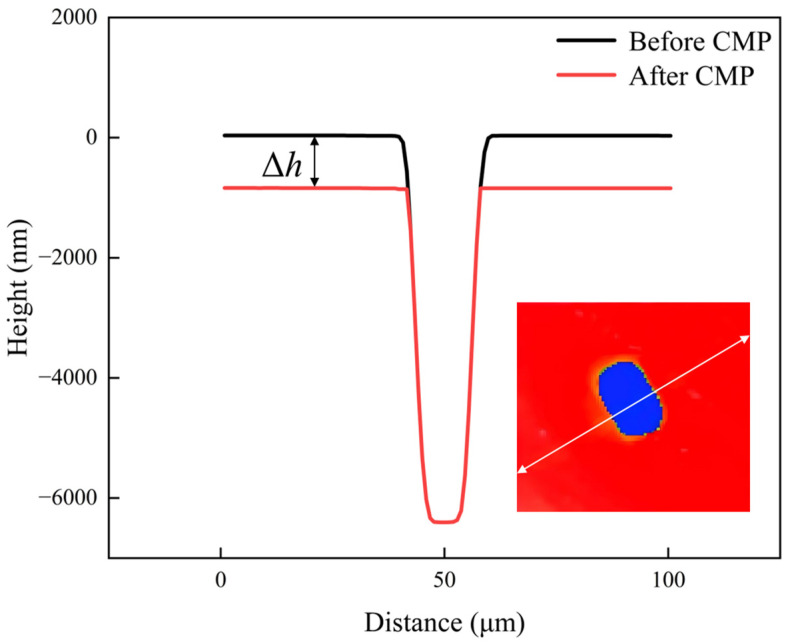
The groove depth difference Δ*h* and the measuring morphology of the groove by Zygo.

Because the removal rate of diamond is extremely low, the traditional weighing method is not suitable for measuring the diamond removal rate. To obtain precise removal rate data, we used a Helios G4 UX Focused Ion Beam (FIB) manufactured via FEI Company (Hillsboro, OR, USA) to prepare grooves with dimensions of approximately 10 µm × 20 µm × 10 µm, with the 1bottom flatness of the groove reaching the nanoscale [41]. As shown in Figure 2, the MRR was determined by measuring the change in groove depth Δ*h*. The MRR values are calculated using Equation (3):

MRR = Δ*h*/t (3)

The MRR values were expressed as the average of the three measurements. A 3D optical surface profilometer Zygo Newview Model 9000 via Zygo Corporation (Middlefield, CT, USA) with a resolution of 0.1 nm was used to accurately measure the groove depth changes and surface topography before and after CMP, with a measurement area of 868 × 868 µm^2^. The insert in Figure 2 displays the measuring morphology of the groove by Zygo after FIB processing and the white line indicates the position and orientation of the measurement. The XPS spectra were performed using an X-ray photoelectron spectrometer ESCALAB Xi + via Thermo Fisher Scientific (Waltham, MA, USA). The peak shape was fitted by Avantage software and calibrated with the C1s peak at 284.8 eV.

## 3. Results and Discussion

**Figure 3:**
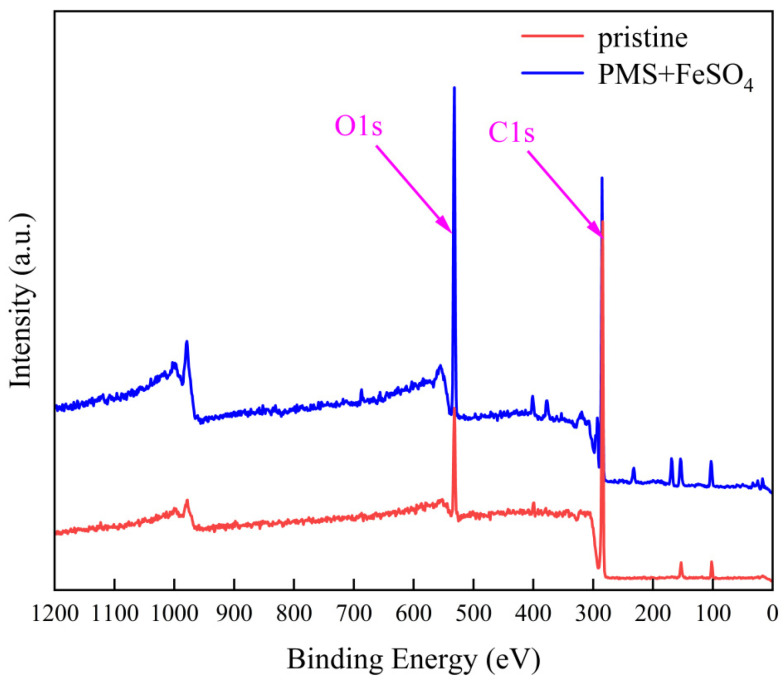
XPS global survey spectra of the diamond surface before and after immersing in PMS + FeSO_4_ slurry for 12 h.

**Figure 4:**
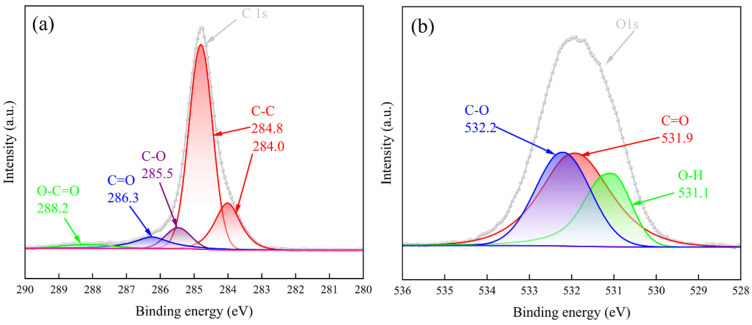
XPS high-resolution survey spectra of (**a**) C1s and (**b**) O1s after oxidizing in PMS + FeSO_4_ slurry.

The XPS results are shown in Figure 3 and Figure 4. The XPS global survey spectra of the SCD surface before and after oxidation are displayed in Figure 3. The two main peaks at 284.8 eV and 531.9 eV correspond to the C1s and O1s energy levels, respectively [42,43]. The intensity of the O1s peak is significantly enhanced after oxidation, indicating an increase in the content of oxygen-containing groups on the SCD surface. Figure 4a,b present the XPS high-resolution survey spectra of C1s and O1s after oxidation. In the C1s spectrum, the dominant peak at 284.8 eV is assigned to the intrinsic sp^3^-hybridized C–C bond of diamond, while the additional peaks at 285.5 eV (C–O), 286.3 eV (C=O), and 288.2 eV (O–C=O) clearly demonstrate the formation of oxygen-containing functional groups on the diamond surface after treatment. Similarly, the O1s spectrum further confirms this oxidation behavior, where the peaks at approximately 532.2 eV (C–O) and 531–531.9 eV (C=O) indicate that oxygen species have been incorporated into the surface structure. These results reveal that the chemically inert diamond surface undergoes remarkable oxidation in the PMS + Fe^2+^ system. This can be attributed to the activation of peroxymonosulfate by ferrous ions, which generates highly reactive SO_4_^−^· and ·OH radicals. With strong oxidizing capability, these radicals can effectively attack the diamond surface, break the stable sp^3^ C-C bonds, and convert surface carbon atoms into oxygen-containing functional groups. As a result, a thin oxidized layer (C–O, C=O, O–C=O) is formed on the outermost surface of diamond.

**Figure 5:**
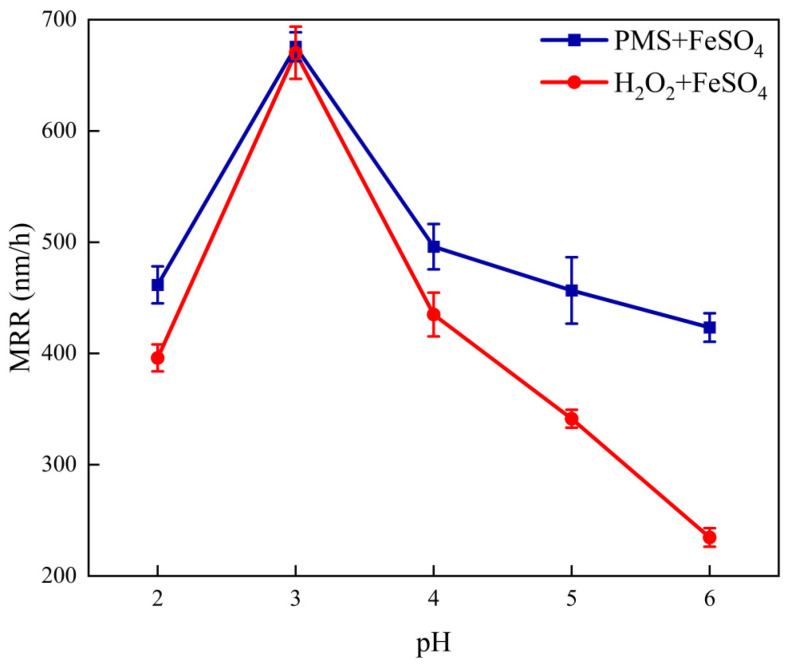
MRR of SCD under different slurry pH conditions using PMS + FeSO_4_ and H_2_O_2_ + FeSO_4_ slurries.

As shown in Figure 5, when using PMS + FeSO_4_ and H_2_O_2_ + FeSO_4_ as the CMP slurry, the MRR exhibits a trend of initially increasing and then decreasing. When PMS + FeSO_4_ is used as the CMP slurry, the MRR reaches a peak of approximately 676 nm/h at pH = 3, which was slightly higher than the MRR of 670 nm/h obtained when H_2_O_2_ + FeSO_4_ was used as the CMP slurry under the same conditions. This is because when H_2_O_2_ is used as the oxidant, the classical Fenton reaction mainly produces ·OH radicals, as described by Equation (4) [44]. In contrast, when PMS serves as the primary oxidant, Fe^2+^ catalyzes the decomposition of PMS, predominantly generating SO_4_^−^· that exhibit a higher oxidation potential and a longer half-life, as shown in Equation (5) [45]. When pH = 3, Fe^2+^ is largely dissolved in the form of highly active free ions, maximizing homogeneous catalytic efficiency [29]. The abundant generation of these two highly reactive radicals can significantly enhance the oxidation efficiency at the reaction interface, thereby accelerating the chemical modification process on the target material surface. Furthermore, from a kinetic perspective, the rate constant for the activation of PMS by Fe^2+^ to produce SO_4_^−^· (approximately 10^4^∼10^5^ M^−1^s^−1^) is higher than that for the activation of H_2_O_2_ to generate ·OH (approximately 50–70 M^−1^s^−1^) [46,47]. Conversely, at an excessively acidic pH = 2, excessive H^+^ may alter radical reaction pathways and suppress the Fe^2+^/Fe^3+^ redox cycle, thereby reducing the effective utilization of reactive species [28]. Notably, the most significant feature of the PMS + FeSO_4_ CMP slurry was its ability to maintain a high removal rate at higher pH values (maintaining high MRRs of approximately 457 nm/h and 423 nm/h at pH = 5 and 6, respectively). In contrast, the traditional Fenton (H_2_O_2_ + FeSO_4_) CMP slurry suffered a catastrophic decline in efficiency when the pH exceeded 4.0, with MRRs dropping to only about 341 nm/h and 235 nm/h at pH = 5 and 6. In the traditional Fenton slurry, when the pH increases above about 4, dissolved iron species tend to hydrolyze and precipitate, which suppresses the catalytic cycle and reduces the effective oxidation efficiency at the polishing interface [44]. However, PMS relies less on homogeneous Fe^2+^ catalysts than H_2_O_2_ and can still generate free radicals after catalyst precipitation [45]. Moreover, with increasing pH, H_2_O_2_ tends to undergo ineffective decomposition into H_2_O and O_2_, as shown in Equation (6) [48]. Additionally, when the pH increases, a portion of the generated SO_4_^−^· can further react with H_2_O or OH^−^ to yield secondary ·OH, thereby establishing a synergistic oxidation system dominated by SO_4_^−^· and supplemented by ·OH. These reaction mechanisms are presented in Equations (7) and (8) [45]. Therefore, in the process of diamond CMP, the system can generate highly oxidizing species more efficiently and promote the oxidation transformation of the diamond surface. This enables the CMP slurry to achieve efficient processing in a very wide process window.

Fe^2+^ + H_2_O_2_ → Fe^3+^ + ·OH + OH^−^ (4)

Fe^2+^ + HSO_5_^−^ → Fe^3+^ + SO_4_^−^· + OH^−^ (5)

2 H_2_O_2_ → H_2_O + O_2_ (6)

SO_4_^−^· + H_2_O → SO_4_^2−^ + ·OH + H^+^ (7)

SO_4_^−^· + OH^−^ → SO_4_^2−^ + ·OH (8)

**Figure 6:**
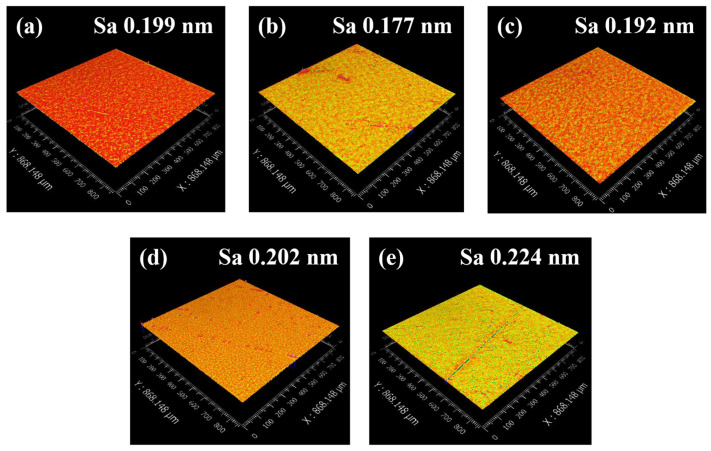
Surface morphology of SCD after CMP with PMS + FeSO_4_ slurry: (**a**) pH = 2; (**b**) pH = 3; (**c**) pH = 4; (**d**) pH = 5; (**e**) pH = 6. (Measuring area: 868 × 868 μm^2^).

Before CMP, the diamond surface typically exhibits severe mechanical damage, including deep grooves, microcracks, and high roughness caused by mechanical pre-grinding. As shown in Figure 6, ultra-smooth surfaces with Sa ≈ 0.2 nm are stably obtained under the condition of pH = 2–6. Within the scanning area of 868 × 868 μm^2^, the surface roughness Sa = 0.177 nm was obtained when pH = 3. This observation confirms that the PMS + FeSO_4_ slurry not only yields a globally flat surface but also ensures exceptional local smoothness, meeting the stringent requirements for advanced diamond-based electronic and optical applications.

**Figure 7:**
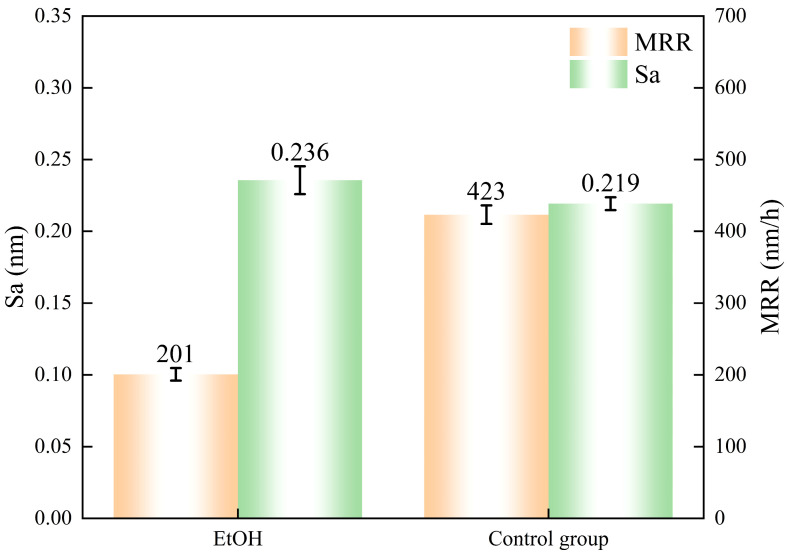
MRR and Sa obtained through the free radical quenching experiment on PMS + FeSO_4_ CMP slurry.

To verify that the PMS + FeSO_4_ slurry can still maintain strong material removal capacity under weakly acidic pH conditions due to the presence of abundant active radicals in the slurry, this study conducted radical quenching experiments on the PMS + FeSO_4_ slurry using EtOH. Figure 7 shows the comparison of the MRR and Sa obtained with and without the addition of ethanol in the PMS + FeSO_4_ slurry. In the absence of ethanol (control group), the CMP slurry exhibits a high MRR of approximately 423 nm/h and a Sa of approximately 0.219 nm. However, after the introduction of EtOH, the MRR decreases sharply by half to about 201 nm/h, which is similar to the MRR obtained by using H_2_O_2_ + FeSO_4_ slurry at pH = 6. Meanwhile, Sa also slightly increases to approximately 0.236 nm. The significant decrease in the MRR and increase in the Sa confirm that the oxidation of the diamond surface is strongly dependent on the free radical species generated during the PMS activation process. The addition of ethanol effectively quenches SO_4_^−^· and ·OH radicals, thereby weakening the oxidation capability of the slurry. Therefore, these results indicate that active radicals still exist in the PMS + FeSO_4_ slurry at pH = 6. This also explains why the PMS + FeSO_4_ slurry can maintain stable polishing performance for SCD in a weakly acidic environment.

**Figure 8:**
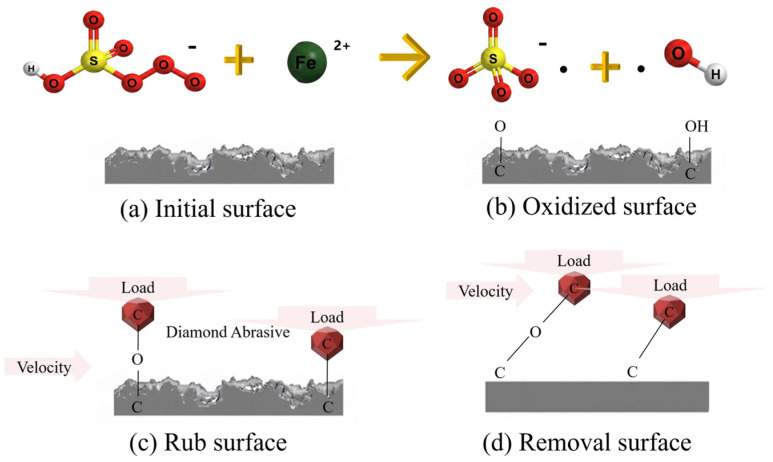
Schematic diagram of carbon atom removal mechanism in the CMP process.

Based on the above experimental results and reasonable speculation, an atomic-level material removal model is proposed in this paper to clarify the stable atomic-level removal mechanism of SCD over a wide pH range of the slurry during CMP, as shown in Figure 8. The transition metal ion (Fe^2+^) efficiently activates PMS, generating a stable and continuous supply of highly reactive SO_4_^−^· and ·OH. These free radicals initiate oxidative attacks on the diamond surface. This continuous bombardment is believed to lead to significant chemical adsorption of oxygen, which may be manifested by the formation of oxygen-containing functional groups (such as C=O and C–OH) on the outermost atomic layer. In the dynamic friction process, the local high contact pressure forces the diamond abrasive particles to contact closely with these oxidation sites. Therefore, it is expected to establish a strong interface bridge bond (C–O–C) to connect the abrasive grain and the modified diamond substrate. According to the literature, the dissociation energy of the C–O bond (~1077 kJ/mol) is significantly larger than that of the bulk C–C bond (~610 kJ/mol) [49]. Therefore, when subjected to the tangential shear stress generated by the rotation of the indenter, the relatively weak underlying C–C bond in the diamond lattice preferentially breaks. This tribochemical tearing effectively removes carbon atoms layer by layer, contributing to atomic-level surface planarization. In addition, strong abrasive shearing can directly mechanically expel the adsorbed oxygen-containing groups. This makes the exposed surface carbon atoms in a highly active and under-coordinated state, which is conducive to the direct formation of transient C–C bonds with the abrasive particles and further promotes atomic detachment. Ultimately, the stable polishing performance exhibited by the PMS + Fe^2+^ slurry over a wide pH range can be attributed to its ability to continuously generate reactive free radicals and drive oxidation in a broad pH window. This continuous chemical action promotes a continuous high density of active C=O and C–OH sites, maximizing the probability of forming key interfacial bridge bonds that control the mechanical exfoliation stage.

## 4. Conclusions

In this study, a PMS-based CMP slurry is developed for the polishing of SCD. The polishing performance is systematically evaluated in terms of material removal rate, surface morphology, and roughness, and the CMP mechanism is further clarified through radical quenching experiments and XPS analysis. The main conclusions are as follows:

(1) The PMS + Fe^2+^ CMP slurry can effectively generate highly reactive radicals, providing strong oxidation capability and enabling efficient material removal over a wide pH range, thereby overcoming the narrow pH limitation of traditional Fenton slurry.
(2) The CMP mechanism is dominated by radical-driven oxidation, in which SO_4_^−^· and ·OH oxidize the diamond surface to form oxygen-containing functional groups (C–O, C=O, O–C=O), weakening C–C bonds and facilitating tribochemical material removal through abrasive interaction, ultimately leading to ultra-smooth surface planarization.
(3) Under optimal conditions, the CMP slurry achieves a maximum MRR of 676 nm/h and produces an ultra-smooth surface with a roughness of Sa = 0.177 nm in the measuring area of 868 × 868 μm^2^, while still maintaining a high MRR above 400 nm/h under weakly acidic conditions, demonstrating favorable polishing stability over a wide pH range.

## Figures and Tables

**Table 1:** Components of different CMP slurry.

Groups	Oxidizing Agent	Catalyst	Abrasive
(a)	5 wt% PMS	0.1 mol/L FeSO_4_	5 wt% diamond powder
(b)	30 wt% H_2_O_2_	0.1 mol/L FeSO_4_	5 wt% diamond powder

## Data Availability

The original contributions presented in this study are included in the article. Further inquiries can be directed to the corresponding author.
